# Optimization of Fatigue Performance of FDM ABS and Nylon Printed Parts

**DOI:** 10.3390/mi14020304

**Published:** 2023-01-24

**Authors:** Andrey Yankin, Gaini Serik, Saniya Danenova, Yerassyl Alipov, Ali Temirgali, Didier Talamona, Asma Perveen

**Affiliations:** Department of Mechanical and Aerospace Engineering, School of Engineering and Digital Sciences, Nazarbayev University, Astana 010000, Kazakhstan

**Keywords:** Fused Deposition Modeling (FDM), acrylonitrile butadiene styrene (ABS), polyamide (Nylon), fatigue, ANCOVA regression analysis, Taguchi analysis, parametric study, numerical study

## Abstract

This research work aims to proceed with the optimization of Fused Deposition Modeling (FDM) printing parameters for acrylonitrile butadiene styrene (ABS) and polyamide (Nylon) to improve fatigue resistance. For that purpose, the methodology of the paper involves two main approaches: experimental study and finite element analysis. The experimental part of the paper used the Taguchi method to find the effects of printing internal geometry, printing speed, and nozzle diameter on the fatigue life of ABS and Nylon plastic materials. ANCOVA multiple linear regression and sensitivity analysis was used to investigate the effects of printing parameters on the fatigue life of materials. The analysis of the results revealed: Nylon performed better than ABS, but had a higher slope; the ‘tri-hexagon’ structure resulted in the highest fatigue life, but the effect was statistically significant only for ABS material; the fatigue life of both materials increased with decreasing the nozzle diameter; the printing speed had no statistically significant influence neither on ABS nor Nylon. The experimental results then were validated by numerical simulations and the difference between the values was within ±14% depending on the experiment. Such differences might occur due to numerical and experimental errors.

## 1. Introduction

There are a variety of different additive manufacturing techniques, one that the object of interest here is fused deposition manufacturing known as FDM. It involves a nozzle that contains molted filament to transform on a 2D plane plate to build up a layer of a cross-sectional region of a digitalized object. Then the platform moves in up or down directions to continue the layer-by-layer construction process [1].

The FDM process involves thermoplastic materials of high strength such as polycarbonate (PC), polylactic acid (PLA), acrylonitrile butadiene styrene (ABS), polyamide (Nylon), etc. [2,3,4,5]. The application of FDM printing is essential when it comes to inexpensive parts in a short period of time, rigid models, and the construction of a prototype for validation purposes. Thus, the areas of application of 3D printed parts using FDM technology and thermoplastic materials are rapidly increasing and currently involve automotive, aerospace, medical, industrial, manufacturing, architecture, etc. [6,7].

In spite of the fact that there are many advantages of FDM printing over conventional methods, the limitations of this technique are varied in range from low-lying mechanical properties to lousy surface quality of printed parts. One of the most significant challenges is associated with voids formation between layers of printed parts [8]. This occurs owing to the weak interaction between the extruded layer and solidified part [9]. Therefore, it is crucial to examine the mechanical properties of finalized printed objects.

The most common process parameters are infill density, infill patterns (internal geometric structure), extrusion temperature, nozzle diameter, layer thickness, raster angle, build orientation printing speed, etc. [5,10,11]. Based on the literature review findings, infill density tends to be one of the primary factors affecting mechanical behavior. For example, the strength properties of the printed ABS, PLA, and Nylon increase as infill density increases [12,13,14,15]. By so doing, this parameter can be varied from 0% (hollow part) to 100% (solid part).

Along with infill density, the internal geometric structure plays a crucial role in the mechanical properties. It defines the interaction between infilled filaments at the moment of being loaded. Some of the commonly used infill patterns are hexagonal, linear, and diamond. It should be noted that one pattern could provide better results for tensile or compressive properties while the same pattern may not hold well for a component subjected to other types of load [16].

Holding under the control of the relationship between nozzle size and layer thickness helps to regulate the air gaps in the structural matrix of the FDM printed part [16]. Increasing the nozzle diameter relative to layer thickness results in higher flexural strength. This is because increasing the ratio of the nozzle to layer thickness produces more contact surfaces between the layers of printed parts [17].

The printing speed parameter is associated with how fast the nozzle and the rest of the transformable parts of a 3D printer move relatively to stationary elements. The target idea of this parameter is to compromise the duration of printing and the quality of printed parts. At too high a printing speed, the model ends up with a weak interaction between extruded layer and solidified part. Moreover, there are still plenty of parameters that could be varied during the manufacturing process. More information about them may be found in some review papers [5,10,18,19].

FDM components are often subjected to cyclic loads, which might be a reason for fatigue failure. It is evident that the fatigue properties also depend on process parameters. For instance, it was illustrated that fill density, nozzle diameter, and layer thickness were the most influential factors in the fatigue life of PLA [20,21]. The optimal filling pattern for the Nylon was investigated in the paper [22]. As a result, specimens with a triangular filling pattern and matrix density of 20%, reinforced with carbon fiber at 0 degrees, showed better fatigue performance.

The effects of the print direction for PLA [23,24], ABS [23,25], and Nylon [26] as well as raster orientation for PLA [27] and ABS [28] components were explored. These works reveal that print direction and raster orientation have a significant effect on the fatigue performance of FDM materials. In addition, Ziemian et al. [29] investigated the characteristics of fatigue damage accumulation using FDM specimens for multiple mesostructure combinations, i.e., fiber orientations and layering patterns.

Some compare studies are presented in articles [30,31,32]. Namely, Miller et al. [32] conducted a comparative analysis of the fatigue performance of three PCU materials with systematically varied hard and soft segment contents, processed by both injection molding and FDM. In [30,31], Terekhina et al. compared the fatigue performance of various polyamides produced by FDM and selective laser sintering.

In the article [33], the authors applied a slightly different approach. At first, they conducted monotonic torsion tests at various infill patterns to obtain the best configuration for ABS. After that, the best parameter configuration was chosen to examine the fatigue properties at fully reversed load-controlled loading conditions. In [34], authors determined the S-N curve constants for ABS at fixed printing parameters. In the end, it is worth noticing some paper reviews of the fatigue behavior of 3D-printed polymers [23,35]. The literature review summary is presented in Table 1.

Though there are scholarly works that focus on examining the fatigue behavior of FDM printed parts, research works are mainly focused on determining the S-N curve for materials and provide vague studies on parametric analysis. As a result, the improvement in fatigue performance through optimization of printing parameters has a poor reflection in scholarly papers. Furthermore, there is much less fatigue research on Nylon compared to other plastics, for example, ABS. Thus, this study is focused on the determination of the optimization factor conditions to improve the fatigue properties of two thermoplastics ABS and Nylon produced by FDM.

## 2. Materials and Methods

This study is focused on two types of thermoplastics for FDM printing such as ABS and PA6 (Nylon). According to the literature review, three parameters were chosen to study the fatigue behavior of the FDM printed parts: nozzle diameter, internal geometric structure, and printing speed. Table 2 indicates the parameters and their levels.

The design of experiments was conducted within the Taguchi method to identify significant parameters and their levels for upgrading the fatigue behavior of the 3D printed part by performing a number of experiments. This approach allows a simple and inexpensive method in the various branches involving minimum experimental runs [36]. Such techniques enable researchers and engineers to decrease the utilization of material used, energy consumption, and environmental pollution impact. The Taguchi L9 Orthogonal Array with three levels for each three selected parameters is shown in Table 3.

**Figure 1:**
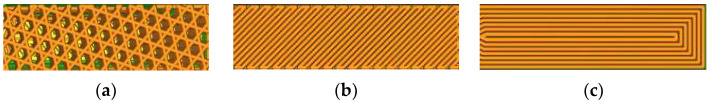
The profiles of internal geometries: Tri-hexagonal (**a**), Zigzag (**b**) and Concentric (**c**).

The parameters for printing were modified using Ultimaker Cura software 4.3 and an ‘STL’ input file with a designated 3D part. Apart from the parameters that were examined, there are constant variables that are summarized in Table 4. The extrusion temperature for ABS was 220 °C and 210 °C for Nylon. The building orientation and internal geometry of a specimen are presented in Figure 1.

**Figure 2:**
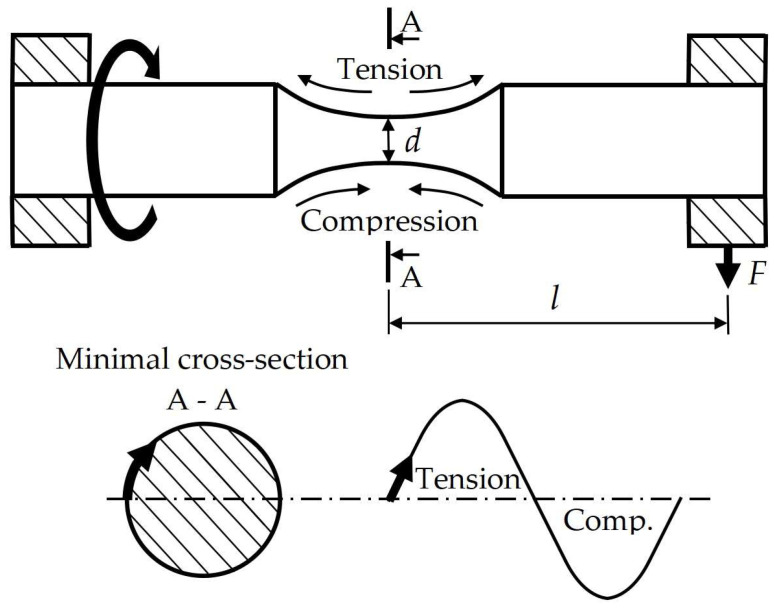
Illustration of stress realization by SM1090V rotating bending fatigue machine.

The experimental procedure involves two main parts: 3D printing and fatigue testing. 3D printing is performed using FDM Ultimaker S3 and S5 printers (Utrecht, Netherlands) whereas fatigue tests are conducted utilizing an SM1090V rotating bending fatigue machine (TecQuipment Ltd. Nottingham, United Kingdom) that can work at frequencies up to 60 Hz. By so doing, the stress ratio was −1. All the applied stress amplitudes were estimated as follows:

(1) $$\sigma_{a}=\frac{32\;l\;F}{\pi d^{3}}$$

where *l* (approximately 28 mm) is the distance from the specimen shoulders where the load was applied to the gauge section, *d* (about 4 mm) is the gauge section diameter, and *F* is the applied force on the shoulders of the specimens (Figure 2).

The observed fatigue results were analyzed quantitatively by fitting the fatigue data in terms of nominal stress amplitude and the number of cycles to failure with a Basquin equation in the form of *σ_a_* = *A*(*N*)*^b^* where *N* is the fatigue life. When the curves are plotted on a log-log scale, they can be represented as regression lines. Then, log(*A*) can be determined as the intercept of the linear fit and its gradient represents *b*.

**Figure 3:**
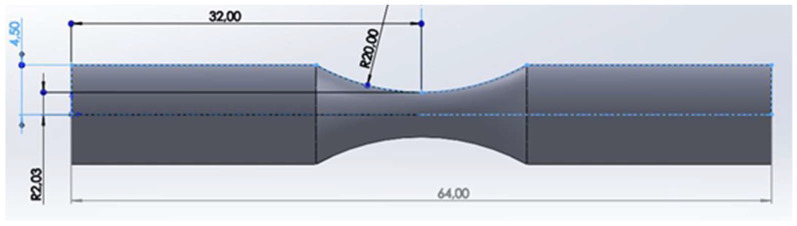
Specimen dimension for fatigue tests.

After the printing process, the dimensions of a part were measured using a caliper because they can be modified after being printed. The hourglass specimen with 64 mm in length and 9 mm in diameter was used to fit the machine dimensions. The geometry is illustrated in Figure 3.

Test results were analyzed using Taguchi analysis. Such a technique is applied to study the effect of nozzle diameter, internal geometric structure, and printing speed on the fatigue life of the materials studied. ANCOVA was used for a multiple regression analysis in which there is at least one quantitative and one categorical variable. And by doing this, the categorical variable with three kinds of internal geometries was re-coded as two new columns (one of the variables was dropped to avoid multi-collinearity) with 0 and 1. The variables were coded 0 for any case that did not match the variable name and 1 for any case that did match the variable name.

**Figure 4:**
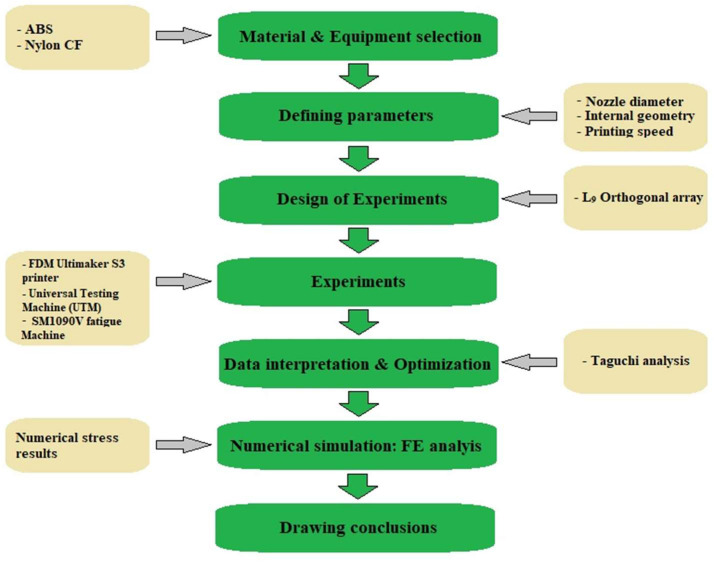
Methodology steps.

During the numerical part of the study, FE analysis was carried out to simulate the test, and simulation results were obtained and compared with experimental results. A numerical study was conducted in Ansys 2021 R2 Workbench static structural analysis. The specimens were designed as a single solid structure. To replicate tests, the specimen was fixed on one end and the force was applied to another end to create the bending. Mesh parameters include 14,268 elements, 61,479 nodes, and an element size of 0.8 mm. The material properties (S-N curves) used in the analysis were taken from work [37]. The methodology steps that were followed during the study are presented in Figure 4.

## 3. Results and Discussion

### 3.1. Experiment

To find the S-N curve, stress was applied by gradually changing the load from 95% to 80% of the ultimate tensile strength (UTS) of materials. In order to obtain UTS, nine tensile tests were conducted for each ABS and Nylon material at different configurations of parameters. The specimen geometry used in tensile tests was designed according to ASTM D638 Type I standard with a working length of 57 mm, work width of 13 mm, and thickness of 3.2 mm. As a result, the mean UTS values were 28.1 ± 2.9 and 50.6 ± 4 MPa for ABS and Nylon, respectively.

**Figure 5:**
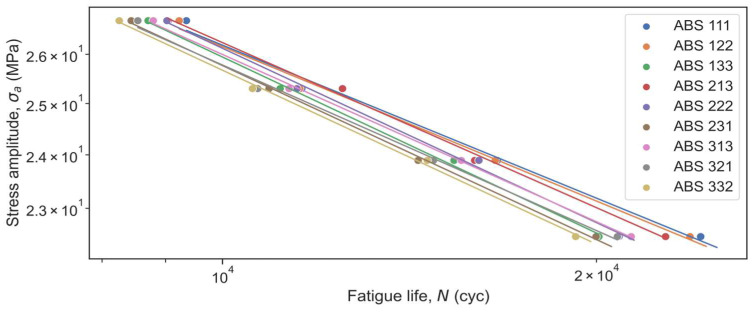
Experimental S-N curve for ABS.

**Figure 6:**
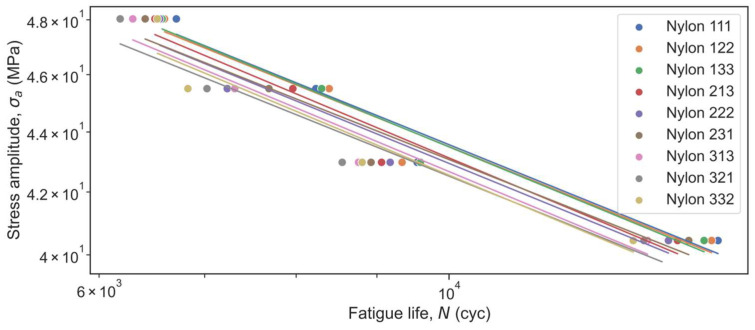
Experimental S-N curve for Nylon.

**Figure 7:**
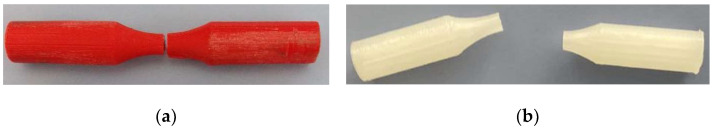
Tested 3D-printed fatigue specimens for ABS (**a**) and Nylon (**b**).

Fatigue test results were obtained in terms of the Stress Amplitude vs. Fatigue Life diagrams (Figure 5 and Figure 6). Overall, the experimental results showed that Nylon performed better than ABS: the fatigue strength of Nylon is higher than that of ABS at the same values of the fatigue life. Specimens made of Nylon material could withstand about 45 MPa load under 9000 cycles, while ABS specimens could withstand about 26.5 MPa under 9000 cycles. However, taking into account the UTS of materials, one can notice a significant lead in the fatigue performance of ABS specimens. Specifically, when 95% load of each material’s UTS was applied, the ABS part could endure 8200–9400 rotations, whereas the Nylon part at its best configuration could not exceed 6720 revolutions. The same trend can be observed for 90%, 85%, and 80% of UTS loads. In addition, fatigue tests showed which configurations have higher fatigue performance and which parameters have a higher influence on results. On average for all loads, for both Nylon and ABS materials, 111 configurations showed the highest results, while 332 and 321 configurations appeared to be the least resistant to fatigue failure. 3D-printed specimens after fatigue tests are presented in Figure 7.

The obtained Basquin parameters were extracted from the plots and reported for each manufacturing condition in Table 5. Smaller absolute *b* values (i.e., reduced slope) represent a higher fatigue performance. As one may see from Table 5, the Nylon material has higher log(*A*) and *b* values than ABS overall. It means that initially Nylon has larger fatigue strength but loses it more intensively than ABS.

### 3.2. Sensitivity Analysis

As discussed in the previous section, the Taguchi method was applied to our experiments to analyze the effect of each printing parameter on the final result without having to execute the full factorial experiment that would have 3 to the power of 3, which is 27 different tests. In Table 6, the mean value of each final result, consisting of the logarithm of the fatigue life for ABS and Nylon, is calculated according to the experiment level of changing parameters. The delta is the difference between the maximum and minimum mean values of the corresponding category. According to this delta value, the ranking was made to conclude this section of the sensitivity analysis. The fatigue tests were carried out at four levels of the stress amplitude. Therefore, for the sensitivity analysis, it was used the average value of the fatigue life as follows:

(2) $$N_{m}=\frac{1}{n}\log\left(\overset{n}{\underset{i=1}{\prod }}N_{i}\right)$$

where *n* is the number of stress amplitude values (*n* = 4).

**Figure 8:**
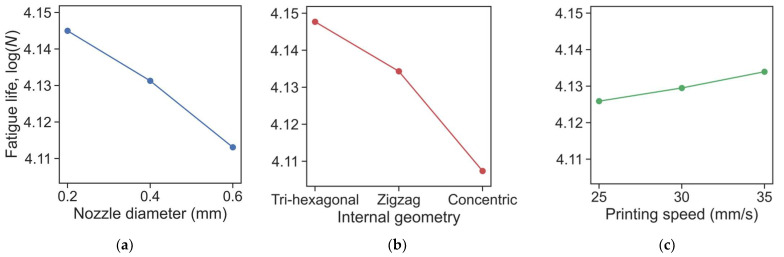

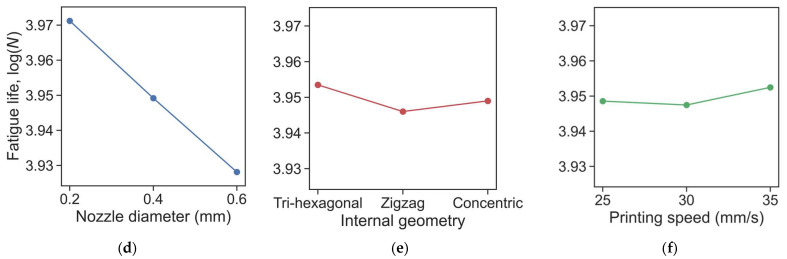
Sensitivity analysis of ABS (**a**–**c**) and Nylon (**d**–**f**) fatigue test for Nozzle diameter (**a**,**d**), Internal geometry (**b**,**e**), and Printing speed (**c**,**f**).

Sensitivity analysis revealed that internal geometry had the highest influence on fatigue performance of FDM printed parts made of ABS whereas it became the second most important parameter for Nylon. As can be seen from Figure 8, ABS with ‘concentric’ shape and Nylon with ‘zigzag’ internal geometries perform in fatigue tests significantly worse by margin than the other two geometries. The ‘tri-hexagon’ structure resulted in the highest fatigue life. This internal structure enables more dense crisscrossed supporting offsets that help layers stick to each other and form support. In the case of the ‘zigzag’ structure, it has less dense crisscrossing offset numbers and thus weaker support. Moreover, walls created by ‘zigzag’ and ‘tri-hexagonal’ infill patterns enable the transfer of a certain portion of the stress to neighboring walls, thus decreasing stress concentrations at local points and delaying crack propagation [33].

Additionally, obtained results proved that a change in nozzle diameter had a notable influence on the continuity of the printed specimen and hence on mechanical properties. An increase in nozzle diameter leads to a reduction of continuity of the internal volume of the part and consecutively to a decrease in fatigue life. Using nozzles with small diameters ensures high density and packing of more material in a certain volume and thus notably reduces the volume of air-filled cavities.

Compared with other printing parameters, the effect of change in printing speed on fatigue performance appears to be much lower. On top of that, from the obtained sensitivity analysis we cannot distinguish a clear pattern or trends associated with printing speed. For different loads and different materials, an increase in printing speed leads to different results without yielding any tendencies. One possible reason for such results might be the small ranges selected for printing speeds [38]. It varied just from 25 to 35 mm/s. Perhaps, the fatigue properties will be more sensitive if a larger speed range were to be chosen, which needs further investigation.

### 3.3. ANCOVA Regression Analysis

In this research study, ANCOVA multiple regression analysis was applied [39]. The model used is the additive one, i.e., it does not take into account any interaction effects. From Table 7 one can see that the model explains 99.2% and 93.2% of the variability in test scores for ABS and for Nylon, respectively (adjusted coefficient of determination, R^2^, is 0.992 and 0.932), and the standard error of estimate represents how far data fall from the regression predictions (fatigue life values in terms of logarithmic scale). Moreover, the F statistic ‘F-value’ of 842.4 and 97.4 with *p*-values of less than 0.001 shows the advantage of this model over an intercept-only model that predicts the average output for all the data.

Reviewing the regression results in Table 7 and Table 8, the models can be represented as:

**ABS:** log(*N*) = 11.39 − 0.028 × Con.geom. + 0.012 × Tri.geom. − 5.20 × log(*σ_a_*) − 0.08 × Noz.d. + 0.0004 × Pr.sp. (3)

**Nylon**: log(*N*) = 11.12 + 0.004 × Tri.geom. − 0.003 × Zig.geom. − 4.34 × log(*σ_a_*) − 0.108 × Noz.d. + 0.0002 × Pr.sp. (4)

The simplified model equations are shown here as follows:

**ABS & Zigzag**: log(*N*) = 11.39 − 5.20 × log(*σ_a_*) − 0.08 × Noz.d. + 0.0004 × Pr.sp. (5)

**ABS & Concentric**: log(*N*) = 11.39 − 0.028 − 5.20 × log(*σ_a_*) − 0.08 × Noz.d. + 0.0004 × Pr.sp. (6)

**ABS & Tri-hexagonal**: log(*N*) = 11.39 + 0.012 − 5.20 × log(*σ_a_*) − 0.08 × Noz.d. + 0.0004 × Pr.sp. (7)

**Nylon & Concentric**: log(*N*) = 11.12 − 4.34 × log(*σ_a_*) −0.108 × Noz.d. + 0.0002 × Pr.sp. (8)

**Nylon & Zigzag**: log(*N*) = 11.12 − 0.003 − 4.34 × log(*σ_a_*) − 0.108 × Noz.d. + 0.0002 × Pr.sp. (9)

**Nylon & Tri-hexagonal**: log(*N*) = 11.12 + 0.004 − 4.34 × log(*σ_a_*) − 0.108 × Noz.d. + 0.0002 × Pr.sp. (10)

From the equations, one can see that the slope of log(*N*) vs. log(*σ_a_*) is −5.20 and −4.34 for ABS and Nylon. Meanwhile, the intercepts of 11.39 (with a 95% confidence interval from 11.15 to 11.62) and 11.12 (with a 95% confidence interval from 10.45 to 11.79) are the expected mean value of log(*N*) of the ABS with the ‘Zigzag’ internal geometry and Nylon with the ‘Concentric’ geometry. The intercept depends on the internal geometry: the ‘Tri-hexagonal’ geometry increases the intercept by 0.012 for ABS and by 0.004 for Nylon whereas the ‘Concentric’ and ‘Zigzag’ geometries decrease it by 0.028 and 0.003 for ABS and Nylon materials, respectively. However, the geometry effect is statistically significant only for ABS material (conc.geom.: *t*-value = −4.9, *p*-value < 0.001; tri-hex.geom.: *t*-value = 2.1, *p*-value = 0.049).

Meanwhile, there is a statistically significant effect of the nozzle diameter on fatigue life: the slope log(*N*) vs. Noz.diam. of −0.080 with *t*-value = −5.8 and *p*-value < 0.001 for ABS as well as −0.108 with *t*-value = −3.2 and *p*-value = 0.003 for Nylon. It means the mean value of log(*N*) decreases by 0.008 and 0.0108 for every 0.1 mm magnification of the nozzle diameter for ABS and Nylon, respectively. On the contrary, the printing speed in the studied range from 24 to 35 mm/s negligibly affects the result for both materials. It should be noted that all the obtained results are in good agreement with the previous section.

In addition, it should be noted that at least 15 tests with 13 degrees of freedom, respectively are necessary according to fatigue standards (for example, PN-H-04325:1976) [40]. In this study, if one considers every S-N curve separately, it looks like four points are not enough to plot a reliable line of best fit because there are only 2 degrees of freedom. However, an application of ANCOVA regression analysis allows for considering test points not separately for every S-N curve but as a single dataset with 36 experiments for an additive regression model (Equation (3) or (4)) with six constants (Table 8), and 30 degrees of freedom. According to the ASTM E-739-91, it is enough for preliminary and exploratory tests [40]. By doing so, one also can calculate the coefficient of determination and the standard error (how far data fall from the predictions, Table 7) that shows the fatigue life variability in terms of logarithmic scale. Thus, the authors assume that 30 tests with 36 degrees of freedom are enough to confirm the trends shown in this study from a statistical viewpoint. In order to use the optimal printing parameters to calculate the fatigue behavior of real structures in the future, the S-N curve constants have to be computed more accurately.

### 3.4. Numerical Simulation

**Figure 9:**
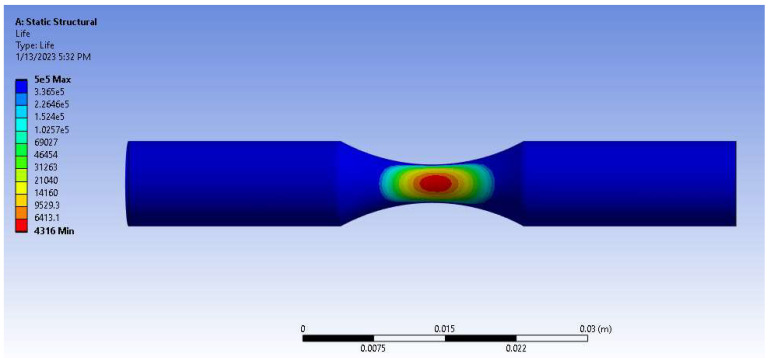
ABS numerical fatigue analysis of fatigue life under 90% UTS load.

**Figure 10:**
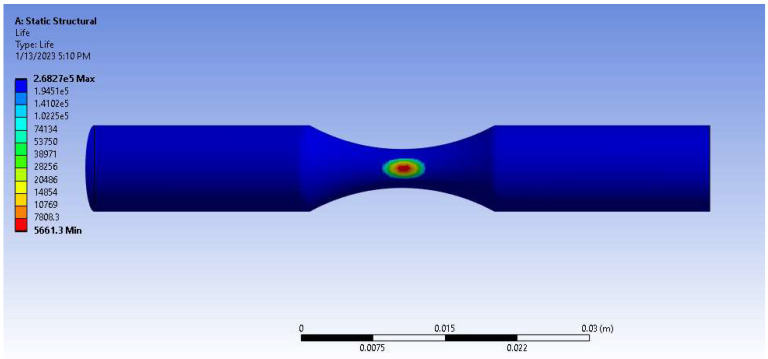
Nylon numerical fatigue analysis of fatigue life under 90% UTS load.

A numerical study for ABS and PA6 (Nylon) plastics is presented in Figure 9 and Figure 10, and Table 9. Comparing the parameters of configured parts with simulation results revealed that the fatigue life of the printed part was on average 11.4% higher than of the numerical one for ABS plastic in terms of logarithmic scale. According to static structural analysis, the ABS part can withstand 10^3.64^ cycles under 90% UTS (25.3 MPa), while experimental results show 10^4.08^ revolutions at the same load level (Figure 9, Table 9). For Nylon material at 90% UTS cyclic load (45.5 MPa), numerical fatigue life reached 10^3.75^ rotations, whereas the experimental one could reach 10^3.92^ cycles (Figure 10, Table 9). The difference might take place due to a number of simplifications and limitations undertaken during numerical analysis. Firstly, simulations consider the specimen as a solid structure. In practice, internal structure plays a significant role in the mechanical properties of the specimen. More realistic models (for instance, layered or laminate models) could provide more accurate simulated results (with differences lower than the reported ones). Moreover, voids, cracks, and internal defects may also notably decrease the performance of the parts during tests. Despite up to 14% differences between numerical and experimental results, for the most part, static structural fatigue analysis showed valid results.

## 4. Conclusions

The parametric study of the printing parameters on the fatigue properties of FDM-printed ABS and Nyon applying Taguchi analysis was developed. In this regard, the work focused on the change in fatigue life due to the change in the printing parameters in a chosen range. The change range of the parameters was based on the conclusion of the review of the literature.

A comparative analysis of the experiments showed that under repetitive tensile and compression load, Nylon performed better than ABS. However, ABS has a lower slope, i.e., fatigue strength decreases less intensively with increasing fatigue life than that for Nylon.

Sensitivity analysis indicated that internal geometry had the highest influence on the fatigue performance of FDM printed parts made of ABS and it became the second most important parameter for Nylon. By so doing, the ‘tri-hexagon’ structure and nozzle diameter of 0.2 mm resulted in the highest fatigue life for both materials. On the contrary, the printing speed parameter in the range of 25–35 mm/s had the least effect on the fatigue life values. The comparison of the experimental and numerical values with theoretical ones showed small differences.

The ANCOVA analysis for multiple linear regression confirmed that ‘Tri-hexagonal’ geometry had a positive effect on fatigue life. However, the effect was statistically significant only for ABS. For Nylon, the change in geometry might be considered negligible. Meanwhile, there is a statistically significant effect of the nozzle diameter on the fatigue life: the mean value of log(*N*) decreases by 0.008 and 0.0108 for every 0.1 mm magnification of the nozzle diameter for ABS and Nylon, respectively. Conversely, the printing speed change in a narrow range (25–35 mm/s) negligibly affects the result for both materials. However, it should be noted that the fatigue properties will probably be more sensitive to a larger speed range.

Thus, the application of results could be served as the optimization factors for better mechanical properties of FDM printed parts for three different types of tests. The findings can serve as reference data for future parametric studies and in the search for a suitable printing configuration for different plastic applications.

## Figures and Tables

**Table 1:** The literature review summary.

Material	Printed Parameters	Results	Refs.
ABS	- nozzle temperature: 220–230 °C - nozzle diameter: 0.5 mm - printing speed: 30 mm/s - layer height: 0.1 mm - infill density: 100%	The average cycle number under the load of 30 N is 3796 cycles. The average cycle number is decreased to 128 cycles when the load is 60 N.	[34]
ABS	- melting temperature: 220 °C - nozzle diameter: 0.5 mm - printing speed: 40 mm/s - layer height: 0.15 mm - infill density: 70%	The parameters for Basquin’s equation that constitutes S-N curve were found with A = 63.31 MPa, and b = −0.204.	[33]
ABS	- nozzle temperature: 245 °C - infill density: 50% - infill pattern: square - printing speed: 60 mm/s - nozzle diameter: 0.4 mm - layer height: 0.15 mm - print direction: horizontal and vertical	The parameters for the Basquin’s equation that constitutes SN curve were found with A = 167.26 MPa, and b = −0.2782 (Horizontal); A = 395.67 MPa, and b = −0.3831 (Vertical)	[23]
ABS	- road width 0.3048 mm - slice height 0.1778 mm - part interior fill style: Solid normal - part fill style: Perimeter/raster - liquefier temperature: 320 °C	The specimens with raster orientation of +45/−45° had the longest fatigue life at each normalized stress level, followed by the 0, 45 and 90° orientations in descending order.	[28]
Nylon	- melting temperature: 195 °C - raster orientation: 0 deg - printing speed: 37.5 mm/s - layer height: 0.2 mm - nozzle diameter: 0.5 mm - number of contours: 3 - filling percentage: 100%	XZ build orientation of Nylon reveals a higher overall fatigue life than that for the XY one	[26]
Nylon CF	- melting temperature: 210 °C - nozzle diameter: 0.3 mm - printing speed: 30 mm/s - layer height: 0.1 mm - infill density: 50% - infill pattern: triangular, hexagonal - filling percentage (%): 20, 50 - filling layers: 24 - walls: 2	The parameters for the Basquin’s equation that constitutes S-N curve were found with A = 206 MPa, and b = −0.039 for specimens with a triangular filling pattern and matrix density of 20%, reinforced with carbon fiber at 0 degrees.	[22]
Nylon (PA12, PA6)	- melting temperature: 250, 195 °C - raster orientation: 0 deg - printing speed: 32, 37.5 mm/s - layer height: 0.2 mm - nozzle diameter: 0.5 mm - number of contours: 3 - filling percentage: 100%	PA12 has a higher overall fatigue life than PA6. The fatigue behavior of PA12 FDM specimens is more resistant than the one obtained by SLS.	[30,31]

**Table 2:** Selected independent variables for fatigue tests.

Parameter	L1	L2	L3
Nozzle diameter	0.2 mm	0.4 mm	0.6 mm
Internal geometry	Tri-hexagonal	Zigzag	Concentric
Printing speed	25 mm/s	30 mm/s	35 mm/s

**Table 3:** L_9_ Orthogonal Array.

Test Code	Nozzle Diameter	Internal Geometry	Printing Speed
111	1	1	1
122	1	2	2
133	1	3	3
213	2	1	3
222	2	2	2
231	2	3	1
313	3	1	3
321	3	2	1
332	3	3	2

**Table 4:** Constant variables for printing.

Parameter	Value
Layer Height	0.15 mm
Orientation	horizontal
Wall Thickness	1.3 mm
Wall Line Count	4
Horizontal Expansion	0 mm
Top/Bottom thickness	1.2 mm
Top Layers	8
Bottom Layers	8
Fan Speed	2%

**Table 5:** Basquin’s parameters.

Test Code	ABS		Nylon	
	log(*A*)	*b*	log(*A*)	*b*
111	2.12 ± 0.05	−0.176 ± 0.012	2.50 ± 0.14	−0.215 ± 0.035
122	2.13 ± 0.04	−0.177 ± 0.010	2.50 ± 0.15	−0.214 ± 0.038
133	2.22 ± 0.03	−0.202 ± 0.007	2.51 ± 0.12	−0.218 ± 0.030
213	2.18 ± 0.03	−0.190 ± 0.008	2.53 ± 0.15	−0.223 ± 0.038
222	2.22 ± 0.04	−0.200 ± 0.009	2.50 ± 0.15	−0.217 ± 0.039
231	2.21 ± 0.02	−0.200 ± 0.005	2.48 ± 0.15	−0.211 ± 0.039
313	2.18 ± 0.02	−0.192 ± 0.004	2.51 ± 0.15	−0.221 ± 0.037
321	2.17 ± 0.04	−0.190 ± 0.009	2.48 ± 0.16	−0.214 ± 0.040
332	2.21 ± 0.03	−0.200 ± 0.006	2.52 ± 0.19	−0.222 ± 0.048

**Table 6:** The comparison of mean values of data according to their categories and ranking of parameters for ABS and Nylon materials.

Changing Parameter	Nozzle Diameter	Internal Geometry	Printing Speed
Level:	The logarithm of fatigue life|ABS		
1	4.1450	4.1478	4.1260
2	4.1313	4.1343	4.1295
3	4.1131	4.1074	4.1340
Delta	0.0319	0.0403	0.0081
**Rank**	**2**	**1**	**3**
Level:	The logarithm of fatigue life|Nylon		
1	3.9712	3.9535	3.9486
2	3.9492	3.9460	3.9475
3	3.9281	3.9490	3.9525
Delta	0.0431	0.0075	0.0050
**Rank**	**1**	**2**	**3**

**Table 7:** Model summary results for fatigue life in terms of logarithmic scale.

Material	R^2^	Adjusted R^2^	Std. Error of Estimate	F-Value	*p*-Value
ABS	0.993	0.992	0.0135	842.4	<0.001 ***
Nylon	0.942	0.932	0.0331	97.40	<0.001 ***

Significance levels: *** *p*-val. ≤ 0.001 (significant), ** *p*-val. ≤ 0.01 (very significant), * *p*-val. ≤ 0.05 (highly significant).

**Table 8:** Multiple linear regression results for fatigue life in terms of logarithmic scale.

Materials	Coefficient	Std. Err.	*t*-Value	*p*-Value	Confidence Interval	
					[0.025	0.975]
ABS						
Const.	11.39	0.11	99.8	<0.001 ***	11.15	11.62
log(*σ_a_*)	−5.20	0.08	−64.2	<0.001 ***	−5.37	−5.04
Conc.geom.	−0.028	0.006	−4.9	<0.001 ***	−0.039	−0.016
Tri-hex.geom.	0.012	0.006	2.1	0.049 *	6.6 × 10^−5^	0.024
Noz.diam.	−0.080	0.014	−5.8	<0.001 ***	−0.108	−0.052
Print.speed	0.0004	0.001	0.7	0.495	−0.001	0.002
**Nylon**						
Const.	11.12	0.33	33.7	<0.001 ***	10.45	11.79
log(*σ_a_*)	−4.34	0.20	−21.8	<0.001 ***	−4.74	−3.93
Tri-hex.geom.	0.004	0.014	0.3	0.761	−0.024	0.032
Zigz.geom.	−0.003	0.014	−0.2	0.845	−0.031	0.025
Noz.diam.	−0.108	0.034	−3.2	0.003 **	−0.177	−0.039
Print.speed	0.0002	0.001	0.1	0.912	−0.003	0.003

Significance levels: *** *p*-val. ≤ 0.001 (significant), ** *p*-val. ≤ 0.01 (very significant), * *p*-val. ≤ 0.05 (highly significant).

**Table 9:** Comparison of experimental results and numerically obtained data.

UTS %	ABS			Nylon		
	log(*N*)		Difference %	log(*N*)		Difference %
	Experiment	Numerical		Experiment	Numerical	
95	3.97	3.56	10.5	3.83	3.30	13.8
90	4.08	3.64	10.9	3.92	3.75	4.2
85	4.22	3.74	11.5	3.98	4.26	−7.1
80	4.39	3.84	12.6	4.17	4.73	−13.4

## Data Availability

The data will be provided by the corresponding author upon request.

## Abbreviations

**Nomenclature**	**Description**
*l*	the distance from the specimen shoulders where the load was applied to the gauge section
*d*	the gauge section diameter
*F*	the applied force on the shoulders of the specimens
*N*	the fatigue life
log(*A*)	the intercept of the linear fit
*b*	the gradient of the linear fit
*σ_a_*	the stress amplitude
UTS	the ultimate tensile strength
FDM	the Fused Deposition Modeling
ABS	acrylonitrile butadiene styrene
Nylon	Polyamide
